# Age: A Moderating Effect on Cerebrospinal Fluid Fibroblast Growth Factor 21 and Cognitive Function

**DOI:** 10.1002/brb3.70784

**Published:** 2025-08-12

**Authors:** Ligang Shan, Ying Tao, Jiubo Fan, Yuyu Zhou, Danyang Zhao, Yanlong Liu, Xiaoli Han, Suriyakala Perumal Chandran, Fan Wang

**Affiliations:** ^1^ Department of Anesthesiology The Second Affiliated Hospital of Xiamen Medical College Xiamen China; ^2^ Faculty of Medicine Lincoln University College Petaling Jaya Malaysia; ^3^ Medical Neurobiology Lab Inner Mongolia Medical University Hohhot China; ^4^ Xiangyang Central Hospital, Affiliated Hospital of Hubei University of Arts and Science Xiangyang China; ^5^ School of Mental Health Wenzhou Medical University Wenzhou China; ^6^ Clinical Nutrition Department Friendship Hospital of Urumqi in Xinjiang Urumqi China; ^7^ Beijing Hui‐Long‐Guan Hospital Peking University Beijing China

**Keywords:** age, cerebrospinal fluid, cognition, fibroblast growth factor 21, moderation

## Abstract

**Background:**

Fibroblast growth factors(FGF)19 subclass related to endocrine metabolism, including FGF19, FGF21, and FGF23, which is associated with cognition. Age can affect its secretion and has been identified as a significant risk factor for cognitive decline. Consequently, age may moderate the impact of the FGF19 subclass on cognitive function. This study aimed to investigate the association among the FGF19 subclass in cerebrospinal fluid (CSF), cognition, and age and further explore the moderating effects of age on cognitive changes related to the FGF19 subclass.

**Methods:**

In this cross‐sectional study, participants were stratified into two groups based on age, namely the ≤34 year old (n = 128) and >34 year old (n = 63) groups, and CSF FGF19 subclass levels were measured. Cognition was assessed using the Montreal Cognitive Assessment Scores.

**Results:**

Age may play a moderating effect in the relationship between CSF FGF21 and cognition (R^2^ = 0.12, β = ‐0.32, *p*  = 0.003). In individuals aged >34 years old, a negative correlation was observed between serum TG levels and MoCA scores (r = ‐0.31, *p* = .041). Contrastingly, no correlation was noted between CSF FGF19 and CSF FGF23 levels with MoCA scores in both groups, respectively (all *p* > 0.05).

**Conclusions:**

This is the first study to report that age plays a moderating effect in the relationship between CSF FGF21 and cognition. Moreover, higher CSF FGF21 levels have a protective effect on cognition in individuals aged ≤34 years old. However, individuals aged>34 years old can improve cognition via alternative strategies.

## Graphical abstract

The effects of CSF FGF21 on cognition differ based on age, as evidenced by higher CSF FGF21 levels only exerting protective effects on cognition in males aged ≤34 years old. On the contrary, there was a trend toward cognitive decline with high CSF FGF21 levels in individuals aged >34 years old.

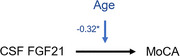

**Abbreviations**
AD: Alzheimer's disease
ALT: alanine aminotransferase
ANCOVA: analysis of covariance
AST: aspartate aminotransferase
BBB: the blood‐brain barrier
BCSFB: the blood‐cerebrospinal fluid barrier
CNS: central nervous system.
CSF: cerebrospinal fluid
FGF: fibroblast growth factors
FGFR1: fibroblast growth factor receptor 1
GGT: gamma‐glutamyltransferase
GLU: Glucose
HDL: high‐density lipoprotein
KLB: β‐Klotho
LDL: low‐density lipoprotein
MoCA: Montreal Cognitive Assessment
PI3K: phosphoinositide 3‐kinase
RBANS: the assessment of neuropsychological status
SD: standard deviation
STROBE: the Strengthening the Reporting of Observational studies in Epidemiology
TC: total cholesterol
TG: triglycerides

## 1 Introduction

As the aging population rises, the prevalence of cognitive decline progressively increases (Branigan and Dotta 2024). Cognitive decline is the earliest manifestation of Alzheimer's disease (AD) (Rabin et al. 2017), which currently affects over five million individuals and is projected to affect 152 million individuals by 2050 (Scheltens et al. 2021). AD burden impacts individuals and their families and imposes a heavy economic burden on society, with estimated global costs reaching US$1 trillion annually (Lane et al. 2018). Therefore, there is a pressing need to elucidate the pathophysiological mechanisms underlying cognitive decline.

Previous research reported that regulatory factors participating in lipid metabolism impact cognitive functions (Wang et al. 2018). The fibroblast growth factor (FGF) 19 subclass, composed of FGF19, FGF21, and FGF23, is implicated in regulating lipid metabolism and infiltrates the brain through the blood‐brain barrier (BBB) or the blood‐cerebrospinal fluid barrier (BCSFB) to bind to receptors, thereby influencing cognitive function (Ursic‐Bedoya et al. 2022; Hsuchou et al. 2013; Do et al. 2024; Ursem et al. 2021; Zhai et al. 2023). Various FGF19 subclasses exert distinct effects on cognitive function. For instance, overexpression of FGF19 significantly increased the number of Nissl bodies in the hippocampus, indicative of neuronal damage alleviation (Lu et al. 2023). In addition, FGF19 can bind to receptors located in brain areas involved in learning and memory, thereby exerting protective effects on cognitive functions (Chen et al. 2023; Miyake and Itoh 1996). FGF21 is ubiquitously expressed in the brain region responsible for spatial learning and memory (Zhou et al. 2022). Meanwhile, FGF21 can enhance hippocampal synaptic plasticity and subsequently delay the onset of cognitive impairment (Sa‐Nguanmoo et al. 2016; Taliyan et al. 2019). FGF23 is present in the hippocampus and amygdala and plays a key regulatory role in cognitive processes (Ursem et al. 2021; Kunert et al. 2017). Overexpression of FGF23 may induce impaired long‐term potentiation in the hippocampus, resulting in cognitive and memory decline (Liu et al. 2011; Drew et al. 2014).

As is well documented, age is a significant risk factor for cognitive decline (Mahncke et al. 2006). The results of an epidemiological investigation revealed that the incidence rate of cognitive function decline is below 4% among young individuals and steadily increases with age to 11% (Gomez‐Soria et al. 2023; Alzheimer's Association 2014). Similarly, research described that the levels of the FGF19 subclass in cerebrospinal fluid (CSF) also fluctuate with age. Notably, a statistically significant positive correlation was noted between CSF FGF21 levels and plasma FGF21 levels in rodents and humans, with the levels increasing over time (Tan et al. 2011; Taliyan et al. 2019; Villarroya et al. 2018). FGF19 and FGF23, the remaining two members of the FGF19 subclass, share structural and phylogenetic overlap with FGF21 (Taliyan et al. 2019; Dolegowska et al. 2019). Consequently, the CSF levels of FGF19 and FGF23 may also increase with advancing age. Thus, age may regulate the levels of the FGF19 subclass in CSF.

Nevertheless, the impact of age on the relationship between the FGF19 subclass and cognitive function remains elusive. Therefore, this study assessed cognitive performance using the Montreal Cognitive Assessment (MoCA) and measured FGF19 subclass levels in CSF to construct a moderation model and investigate the moderating effect of age on the relationship between the FGF19 subclass and cognitive performance to lay a theoretical reference for preventing and mitigating cognitive decline.

## 2 Materials and Methods

### 2.1 Study Design

A cross‐sectional study was conducted following the Strengthening the Reporting of Observational Studies in Epidemiology (STROBE) statements (https://www.strobe‐statement.org/). The cases consisted of participants who were g34 years old, while the controls comprised individuals who were >34 years old and who were matched to the cases.

### 2.2 Participants

According to earlier studies, estrogen affects cognitive function (Bortz et al. 2022). In addition, men typically engage in regular exercise more frequently than women (Mao et al. 2020), with the rate of anterior cruciate ligament injury being significantly higher in males compared to females. Therefore, to mitigate confounding factors, 191 Chinese male participants scheduled for anterior cruciate ligament reconstruction surgery between September 2014 and January 2016 were recruited in this study. A sudden change in proteomics after 34 years old has been observed (Lehallier et al. 2019); thereby, participants were divided into two groups, namely the age ≤34 years and age >34 years groups. The mean age of participants was 31.76 ± 10.22 years, with 128 participants in the ≤34‐year‐old group and 63 participants in the > 34‐year‐old group.

Sociodemographic data, encompassing age, years of education, and BMI, were collected from participants. Clinical data, such as a history of substance abuse, dependence, and smoking status, were obtained through self‐report and further confirmed by the next of kin and family members. Participants with the following criteria were excluded from this study: (1) a family history of psychosis or neurological diseases, and (2) the presence of systemic or central nervous system diseases, as well as sleep disorders, as determined by the Mini‐International Neuropsychiatric Interview.

All recruited participants had no history of substance abuse or dependence, except for cigarette smoking, and had no other psychiatric disorders based on the criteria defined in the Diagnostic and Statistical Manual of Mental Disorders, fourth edition, which was in use before the publication of the fifth Chinese version. Additionally, none of the participants reported any history of sleep disorders and had not been diagnosed with such conditions.

### 2.3 Assessments, Biological Sample Collection, and Laboratory Tests

The levels of triglycerides (TG), gamma‐glutamyltransferase (GGT), alanine aminotransferase (ALT), aspartate aminotransferase (AST), low‐density lipoprotein (LDL), high‐density lipoprotein (HDL), total cholesterol (TC), and glucose (GLU) were recorded to assess physical condition at admission. These peripheral blood metabolic markers were measured in the morning after overnight fasting in the hospital using a biochemical analyzer (HITACH 7600, Hitachi, Tokyo, Japan).

MoCA, a widely used and simple screening tool for detecting cognitive impairment, was administered to all subjects (Nasreddine et al. 2005). It evaluates eight cognitive domains, encompassing visual‐spatial and executive function, naming, memory, attention, language, abstraction, delayed recall, and orientation. The total MoCA score is derived by adding the points from each successfully completed task and ranges between 0 and 30 points, with higher MoCA scores reflecting better cognitive function. A score below 26 points indicates mild cognitive impairment (Nasreddine et al. 2005). One point was added to the total MoCA score to adjust for the effects of education for subjects with 12 or fewer years of education (McDicken et al. 2019). The sensitivity of the MoCA was 83.8% for cognitive impairments in China (Lu et al. 2011).

As part of the clinical routine for patients undergoing anterior cruciate ligament reconstructive surgery, lumbar puncture was conducted by a licensed anesthetist in the morning prior to the surgical intervention. A 5 mL CSF sample was collected intrathecally. The time interval between the cognitive assessments and lumbar puncture was less than 24 h. Subsequently, each CSF sample was divided into ten 0.5 mL aliquots and promptly stored at ‐80°C for the ensuing experiments. The current optimal protocol for the collection and storage of plasma/cerebrospinal fluid samples includes aliquoting and storing samples at − 80°C to avoid freeze‐thaw cycles leading to cytokine degradation. The interval between hospitalization and surgery did not exceed five days. The CSF levels of FGF19, FGF21, and FGF23 were determined (Cat. SEA746Hu, SCC917Hu, and SEC918Hu, Cloud‐clone Corp., Katy, TX, USA) using ELISA kits with a detection range of 15.6‐1,000 pg/mL. Laboratory technicians were blinded to the clinical data of participants.

### 2.4 Statistical Analysis

All statistical analyses were performed using IBM SPSS Statistics for Windows, Version 22.0 (IBM Corp., Armonk, NY, USA). Figures were generated using GraphPad Prism version 9 (GraphPad Software Inc.).

In this study, part of CSF FGF21 and FGF23 data was previously reported (Liu et al. 2017; Zheng et al. 2024; Li et al. 2018). It is important to note that the present analysis employs a different methodological approach, which has led to the detection of new positive results. Therefore, we would like to acknowledge the use of this variable's data from the previous study and clarify that the present findings are based on an independent and distinct analytical methodology.

The Kolmogorov‐Smirnov normality test and Levene's test of homogeneity were applied to all continuous variables. Given that only the distributions of TC and LDL were normal with homogeneous variances (all *p* > 0.05), ANOVA was used to compare differences between the two groups. The remaining demographic and clinical data were compared using the Mann–Whitney U test. Analysis of covariance (ANCOVA) was used to evaluate differences between groups for CSF levels of the FGF19 subclass and MoCA scores, with BMI, education, and smoking status as covariates. Continuous data were described as mean ± standard deviation (SD), while categorical variables were expressed as frequencies and percentages. Secondly, Spearman correlation analysis was performed to examine the relationships among continuous variables, with BMI, education, and smoking status as covariates.

Thirdly, hierarchical multiple regression analyses were performed to examine the main effects of age group, CSF FGF21 levels, and their interaction on MoCA scores. In statistical models, the moderation effect is implemented through an interaction term. For each regression, BMI, education, and smoking status were initially entered as control variables; the main effects of CSF FGF21 and age group (≤34 years old or >34 years old) were entered in the second step; and the two‐way interaction term (CSF FGF21×age group) was entered in the third step. Finally, simple slope analyses were performed to further identify significant interaction effects.

A two‐sided *p* < 0.05 was considered statistically significant.

## 3 Results

### 3.1 Demographic and Clinical Characteristics

Compared to individuals aged ≤34 years, those aged >34 years had significantly fewer years of education (13.02±2.68 vs. 11.69±3.19, *p* = 0.007), lower ALT (32.75±23.64 vs. 26.49±20.46, *p* = 0.026) and AST levels (21.85±8.83 vs. 19.56±7.62, *p* = 0.044), as well as higher LDL (2.57±0.72 vs. 2.82±0.55, *p* = 0.023), TC (4.64±0.96 vs. 4.95±0.72, *p* = 0.025), and GLU levels (5.12±0.49 vs. 5.35±0.45, *p* = 0.001). The remaining sociodemographic and clinical characteristics were comparable between the two groups (all *p *> 0.05, Table 1).

**TABLE 1:** Differences in demographic and clinical characteristics between the ≤34‐year‐old and >34‐year‐old groups (n = 191).

Variables	≤34 years old (n = 128)	>34 years old (n = 63)	F/χ^2^/z	*p*
	Mean ± SD/n (%)	Mean ± SD/n (%)		
Age (years)	25.81±5.12	43.84±6.71	0.00	<0.001***
BMI (kg/m^2^)	25.40±4.25	25.20±2.98	3971.00	0.934
Education (years)	13.02±2.68	11.69±3.19	2836.50	0.007**
Smoking status			3.80	0.051
Yes	52 (40.6%)	35 (55.6%)		
No	76 (59.4%)	28 (44.4%)		
TG (mmol/L)	1.82±1.23	1.80±1.03	3811.50	0.539
GGT (U/L)	44.46±42.59	41.57±27.35	3719.00	0.537
ALT (U/L)	32.75±23.64	26.49±20.46	3234.00	0.026*
AST (U/L)	21.85±8.83	19.56±7.62	3311.00	0.044*
LDL (mmol/L)	2.57±0.72	2.82±0.55	5.30	0.023*
HDL (mmol/L)	1.27±0.34	1.22±0.23	3199.00	0.826
TC (mmol/L)	4.64±0.96	4.95±0.72	5.14	0.025*
GLU (mmol/L)	5.12±0.49	5.35±0.45	2882.00	0.001**
CSF FGF19 (pg/mL)	315.51±265.70	367.00±254.40	0.38	0.539
CSF FGF21 (pg/mL)	161.06±48.09	175.32±45.82	0.71	0.402
CSF FGF23 (pg/mL)	28.53±14.95	33.13±24.76	3.82	0.052
MoCA scores	26.34±2.23	25.64±30.05	0.97	0.326

*Note*: Data were presented as mean ± SD for continuous variables and as frequencies and percentages for categorical variables.* *p* < 0.05, **p < 0.01, ***p < 0.001.

Abbreviations: ALT, alanine aminotransferase; AST, aspartate aminotransferase; BMI, body mass index; CSF, cerebrospinal fluid; FGF19, fibroblast growth factor 19; FGF21, fibroblast growth factor 21; FGF23, fibroblast growth factor 23; GGT, gamma‐glutamyltransferase; GLU, glucose; HDL, high‐density lipoprotein; LDL, low‐density lipoprotein; MoCA, Montreal Cognitive Assessment; SD, standard deviation; TC, total cholesterol; TG, triglyceride.

### 3.2 Differences and Correlation Analysis of CSF FGF19 Subclass

The levels of CSF FGF19 subclass and MoCA scores were compared as continuous variables using ANCOVA between individuals aged ≤34 years and those aged >34 years, with BMI, education, and smoking status serving as covariates. No significant differences were observed in CSF FGF19 subclass and MoCA scores between the two groups (all *p *> 0.05, Table 1).

**FIGURE 1:**
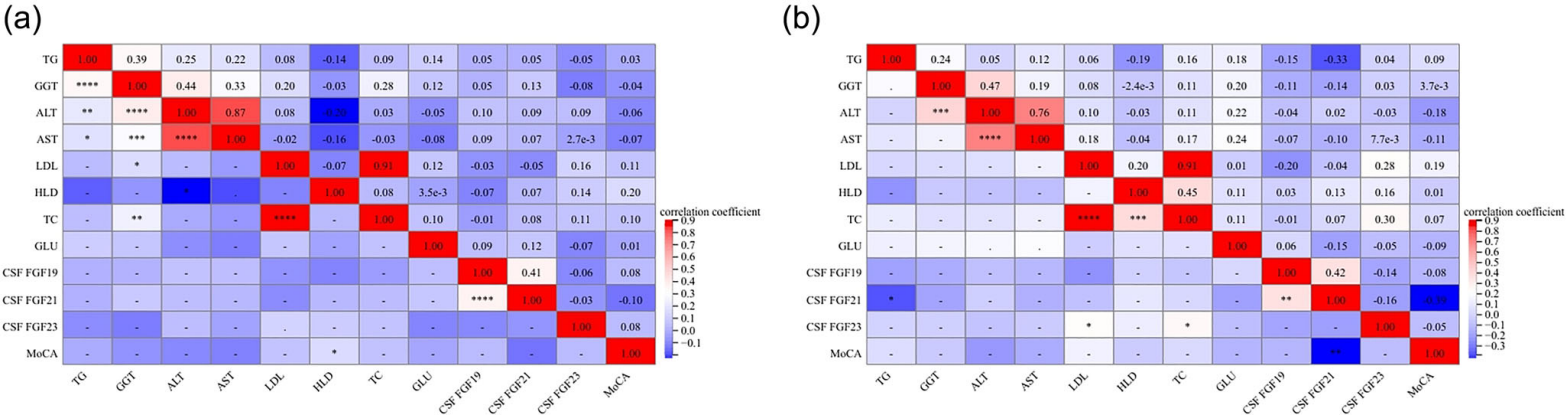
Correlation of general demographic variables, clinical characteristics, cerebrospinal fluid (CSF) fibroblast growth factors (FGF) 19 subclass, and Montreal Cognitive Assessment (MoCA) scores between ≤34‐year‐old and >34‐year‐old groups. (A) Correlation matrix for the study variables in the ≤34‐year‐old group using Pearson's correlation analysis. (B) Correlation matrix for the study variables in the >34‐year‐old group using Pearson's correlation analysis.

Furthermore, partial correlation analysis was performed to assess the correlation of CSF FGF19 subclass and MoCA scores, with BMI, education, and smoking status as covariates. On the one hand, a significantly negative correlation was noted between serum TG levels and MoCA scores in individuals aged >34 years (r = ‐0.31, *p* = 0.041). On the other hand, no correlation was detected between the CSF FGF19 subclass and MoCA scores in both groups, respectively (all *p* > 0.05, Table 2 and Figure 1).

**TABLE 2:** Correlation analysis between baseline characteristics.

≤34 years old >34 years old	TG	GGT	ALT	AST	LDL	HDL	TC	GLU	CSF FGF19	CSF FGF21	CSF FGF23	MoCA scores
TG	—	0.12	0.15	0.13	0.01	−0.06	0.08	0.06	0.05	−0.07	−0.03	−0.02
GGT	0.09	—	0.34	0.32**	0.23*	0.10	0.29	−0.03	0.03	−0.08	−0.11	−0.15
ALT	0.06	0.24	—	0.85	0.08	−0.10	0.06	−0.04	0.18	−0.01	0.02	−0.10
AST	0.03***	0.01	0.85	—	−0.03	−0.07	−0.12	−0.04	0.11	0.07	−0.07	−0.10
LDL	−0.22	−0.03	0.19	0.11	—	−0.12	0.88***	−0.14	0.00	−0.26**	−0.02	0.10
HDL	−0.15	0.01	0.05	0.06	0.13	—	0.04	0.04	0.19	0.19	0.13	0.17
TC	−0.11	0.05	0.30*	0.20	0.89***	0.42**	—	−0.15	0.03	−0.17	−0.05	0.05
GLU	0.22	0.21	0.17	0.13	−0.27	0.01	−0.18	—	0.07	0.07	−0.01	−0.07
CSF FGF19	−0.06	0.23	0.26	0.17	0.05	0.08	0.22	0.14	—	0.19	0.01	0.10
CSF FGF21	−0.21	0.17	0.21	0.11	0.27	0.20	0.35*	−0.36*	0.22	—	0.02	−0.17
CSF FGF23	0.12	0.00	0.01	0.02	0.33*	0.14	0.33*	−0.03	−0.11	0.03	—	0.05
MoCA scores	−0.31*	−0.20	−0.27	−0.21	−0.02	−0.08	−0.18	−0.22	0.06	−0.16	−0.21	—

*Note*: All data are reported as partial correlation analyses, with BMI, education, and smoking status as covariates. * *p* < 0.05, **p < 0.01, ***p < 0.001.

Abbreviations: ALT, alanine aminotransferase; AST, aspartate aminotransferase; CSF, cerebrospinal fluid; FGF19, fibroblast growth factor 19; FGF21, fibroblast growth factor 21; FGF23, fibroblast growth factor 23; GGT, gamma‐glutamyltransferase; GLU, glucose; HDL, high‐density lipoprotein; LDL, low‐density lipoprotein; MoCA, Montreal Cognitive Assessment; TC, total cholesterol; TG, triglyceride.

### 3.3 Moderation Analysis

Firstly, all continuous variables were standardized. Using MoCA scores as the dependent variable, BMI, education, and smoking status were incorporated as covariates in model 1. Model 2 incorporated CSF FGF21 as the independent variable and the age group as the moderating variable, based on Model 1. Model 3 added the interaction term (CSF FGF21 × age group) to Model 2.

**FIGURE 2:**
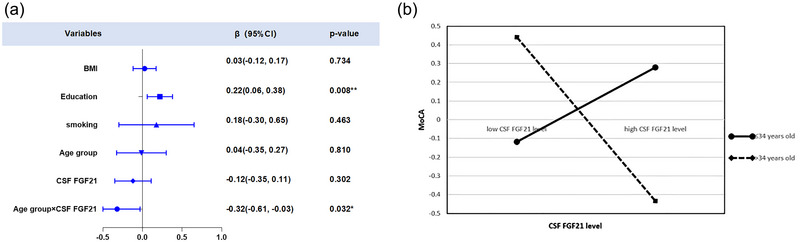
(A) Linear regression analysis displaying the relationship between CSF FGF21 levels and MoCA scores, adjusted for BMI, years of education, smoking status, age group, CSF FGF21, and the interaction between age group and CSF FGF21 (R^2^ = 0.12, β = ‐0.32, t = ‐2.17, *p* = .032). (B) Simple slope analysis depicting the moderating effect of age on the relationship between FGF21 and MoCA scores.

As presented in Table 3, the results from Model 3 revealed a significant positive effect of education on MoCA scores (β = 0.22, t = 2.68, *p* < 0.01). Conversely, the interaction term (CSF FGF21 × age group) was negatively correlated with MoCA scores (β = ‐0.32, t = ‐2.17, *p* < 0.05), as illustrated in Figure 2A.

**TABLE 3:** Linear regression table for the moderation analysis.

	Model 1(MoCA)				Model 2(MoCA)				Model 3(MoCA)			
	β	t	*p*	95%CI	β	t	*p*	95%CI	β	t	*p*	95%CI
BMI (kg/m^2^)	0.04	0.46	0.647	(‐0.11, 0.18)	0.04	0.53	0.594	(‐0.11, 0.19)	0.03	0.34	0.734	(‐0.12, 0.17)
Education (years)	0.21	2.67	0.008**	(0.06, 0.37)	0.21	2.60	0.010*	(0.05, 0.37)	0.22	2.68	0.008**	(0.06, 0.38)
Smoking status	−0.22	−1.43	0.156	(‐0.51, 0.08)	0.13	0.56	0.580	(‐0.34, 0.61)	0.18	0.74	0.463	(‐0.30, 0.65)
Age group	—	—	—	—	−0.08	−0.49	0.624	(‐0.39, 0.24)	0.04	−0.24	0.810	(‐0.35, 0.27)
CSF FGF21 (pg/mL)	—	—	—	—	−0.20	−1.82	0.071	(‐0.42, 0.02)	−0.12	−1.04	0.302	(‐0.35, 0.11)
Age group×CSF FGF21	—	—	—	—	—	—	—	—	**−0.32**	−2.17	0.032*	(‐0.61, ‐0.03)
R^2^	0.07				0.09				0.12			
Adjusted R^2^	0.05				0.06				0.08			
F	4.03				3.18				3.49			
*p*	0.008**				0.009**				0.003**			

*Note*: Model 1: adjusted for BMI, education, and smoking status. Model 2: Model 1 plus adjustment for age group and CSF FGF21. Model 3: Model 2 plus adjusted for the interactions between age group and CSF FGF21. Data are reported as moderation analyses. *p < 0.05, **p < 0.01.

Abbreviations: BMI, body mass index; CSF, cerebrospinal fluid; FGF21, fibroblast growth factor 21; MoCA, Montreal Cognitive Assessment.

To further explore the regulatory role of age group in the relationship between CSF FGF21 levels and MoCA scores, simple slope analysis was carried out, and effect maps were plotted. MoCA scores gradually increased with increasing CSF FGF21 levels in individuals aged ≤34 years. In comparison, MoCA scores progressively decreased with increasing CSF FGF21 levels in individuals aged >34 years (Figure 2B).

## 4 Discussion

To the best of our knowledge, this is the first cross‐sectional study to analyze the moderating effect of age on the relationship between the FGF19 subclass and cognitive function using CSF samples. The primary finding of this study was that the effects of CSF FGF21 on cognition differ based on age, as evidenced by higher CSF FGF21 levels only exerting protective effects on cognition in males aged ≤34 years. On the contrary, there was a trend toward cognitive decline with high CSF FGF21 levels in individuals aged >34 years old. This finding may be attributed to a state of “FGF21 resistance” (Markan 2018).

FGF21 is an endocrine‐regulating factor largely synthesized in the liver and adipose tissues (Falamarzi et al. 2022). Peripheral FGF21 can cross the BBB into the CSF and is considered a crucial link between peripheral metabolic tissues and the brain (Tan et al. 2011; Liu et al. 2017). Several studies have concluded that FGF21 exerts protective effects against nerve injury and cognitive impairment (Sa‐Nguanmoo et al. 2016). Besides, FGF21 can enhance hippocampal synaptic plasticity, increase the density of dendritic spines, restore the function of brain mitochondria, and enhance cognitive function (Sa‐Nguanmoo et al. 2016). It also modulates the astrocyte‐neuron lactate shuttle system to attenuate brain metabolic defects and amyloid β‐induced cytotoxicity, thus acting as a neuroprotective agent in AD (Sun et al. 2020; Taliyan et al. 2019). Previous studies found statistically significant positive correlations between FGF21 levels and cognition, with FGF21 exerting protective effects on cognitive function (Zhang et al. 2024; Tang et al. 2023). However, herein, the protective effect of FGF21 on cognition is not universally applicable and varies with age.

In neonatal brain injury, FGF21 may play a protective role by activating the phosphoinositide 3‐kinase (PI3K) signaling pathway to promote neuronal survival (Ye et al. 2019). Of note, this pathway mediates learning and memory functions in the hippocampus (Zhao et al. 2022). In addition, a study investigating young depressive patients identified a positive correlation between serum FGF21 levels and immediate memory scores during cognitive assessment (Tang et al. 2023). Given that peripheral FGF21 can cross through the BBB and the statistically significant positive correlation between human CSF levels and plasma levels of FGF21 (Tan et al. 2011), we speculated that the CSF levels of FGF21 are also positively correlated with immediate memory in younger people.

However, FGF21 levels in the peripheral circulation increase with aging. Therefore, peripheral FGF21, which crosses through the BBB and enters the CSF, may lead to decreased binding with KLB, leading to FGF21 accumulation in the brain and a state of “FGF21 resistance,” wherein its neuroprotective function is compromised (Minami et al. 2024). The term “FGF21 resistance” refers to a state where circulating or CSF FGF21 levels are increased, accompanied by a decrease in the expression of the FGF21‐receptor complex (Markan 2018). FGF21 exerts its effect through a receptor complex composed of fibroblast growth factor receptor 1 (FGFR1) and the coreceptor β‐klotho (KLB), which activates downstream genes responsible for its protective cognitive functions (Yan et al. 2021). Noteworthily, the coreceptor KLB in the brain is generally expressed in hippocampal neurons (Ananya et al. 2023). The hippocampus is a region critical for learning and memory functions (Babcock et al. 2021). KLB can inhibit the formation of β‐amyloid plaques in the hippocampus, thereby preventing and protecting neurons from their deleterious effects (Ananya et al. 2023). Hippocampal volume is also well‐documented to decline with normal aging, which is considered a hallmark of AD (Raz et al. 2005; Jack et al. 1999). An earlier study pointed out that lower MoCA scores on cognitive assessment measures were associated with smaller hippocampal volumes in AD patients (O'Shea et al. 2016). Importantly, hippocampal atrophy down‐regulates the expression of KLB (Ding et al. 2012). At the same time, the levels of KLB in the CSF decrease with aging, consistent with the results of a clinical survey that demonstrated that CSF KLB levels were significantly lower in elderly patients compared to younger patients (Tippen et al. 2021; Semba et al. 2014). Thus, higher CSF FGF21 levels were associated with low levels of cognitive function, particularly in individuals aged >34 years. This finding may be ascribed to a state of “FGF21 resistance” that develops with age.

The secondary findings included a negative correlation between serum TG levels and MoCA scores in individuals aged >34 years. TGs are simple lipids involved in the storage and transport of energy. Over time, serum TG levels increase, whereas their clearance rate decreases in humans and rodents (Lin et al. 2021). Consequently, serum TG levels are higher in older adults compared to younger adults (Cassader et al. 1996). Animal studies evinced that radioactive TG can cross the BBB into CSF in mice. Moreover, TG was also detected in human CSF (Banks et al. 2018). Numerous studies uncovered that TG contributed to cognitive decline by impairing the maintenance of N‐methyl‐d‐aspartate and that lowering TG levels could reverse cognitive impairment in mice (Farr et al. 2008). Clinical studies also identified a correlation between high serum TG levels and the risk of cognitive impairment. Meanwhile, a case‐control study enrolling 112 subjects with mild cognitive impairment in China identified a correlation between high TG levels and MCI (He et al. 2016). Therefore, reducing peripheral TG levels is paramount for maintaining cognitive function.

However, this study did not identify a correlation between the CSF levels of the remaining two members of the FGF19 subfamily, FGF19 and FGF23, and cognition. Previous studies established that the correlation between FGF19 levels in peripheral circulation and different aspects of cognitive function varies. This may account for the lack of correlation between CSF FGF19 levels and MoCA scores herein. A study used the assessment of neuropsychological status (RBANS) to evaluate cognitive function in patients with depression and determined that serum FGF19 levels were positively correlated with immediate memory, negatively correlated with language function, and not correlated with visual space, attention, and delayed memory (Tang et al. 2023). Furthermore, no correlation was identified between FGF19 levels and the RBANS scale in the normal control group compared to depressed patients (Tang et al. 2023). Compared to RBANS, MoCA is more suitable for the preliminary screening of cognitive function (Nasreddine et al. 2005) and better meets the objectives of this study. In addition, previous studies have principally measured FGF19 levels in peripheral blood, whereas this study detected FGF19 levels in CSF, which may also account for the lack of correlation between CSF FGF19 levels and cognition herein. FGF23 levels increase with decreasing kidney function, with high FGF23 levels associated with cognitive impairment (Drew et al. 2020, Drew et al. 2020). Similarly, high FGF23 levels in the central nervous system (CNS) are correlated with impulsive behavior and poor cognitive performance in hemodialysis patients (Li et al. 2018; Drew et al. 2014). Another study found that FGF23 directly impacts hippocampal neurons and may impair learning and memory function in chronic kidney disease patients (Seidel et al. 2014), implying an association between high FGF23 levels and cognitive impairment. Participants in the present study did not suffer from renal disorders, potentially accounting for the lack of correlation between CSF FGF23 levels and cognition.

Nonetheless, some limitations of the present study cannot be overlooked. Firstly, although females were not recruited to expand the sample size, this decision was made to minimize bias, given that estrogen can increase the production of FGF21 in the liver, and FGF21 levels fluctuate with the menstrual cycle (Allard et al. 2019; Taniguchi et al. 2024). In addition, sex differences in gene expression are abundant in early brain development and persist throughout life (Benoit‐Pilven et al. 2025). Secondly, a considerable number of young people were included in the current study to avoid the emergence of confounding factors associated with chronic diseases that may affect the levels of the CSF FGF19 subclass and cognitive function in elderly individuals (Yan et al. 2021; Xia et al. 2018). Thirdly, although MoCA may not capture domain‐specific cognitive changes, it is a widely used and simple screening tool for detecting cognitive impairment in the general population, and it can evaluate cognitive function across several dimensions, including memory, executive function, and attention, among others (Nasreddine et al. 2005). Future research will analyze the different dimensions of cognitive function in a larger general population. Lastly, as a cross‐sectional study, causality cannot be determined between CSF FGF21 levels and cognitive function. Longitudinal or interventional studies are needed in the future.

## 5 Conclusion

This study revealed that age played a moderating effect on the relationship between CSF FGF21 and cognition, with higher CSF FGF21 levels exerting protective effects on cognition in individuals aged ≤34 years. However, individuals aged>34 years old can improve cognitive function through alternative strategies.

## Author Contributions

**Ligang Shan**: funding acquisition, writing–original draft, investigation, data curation, writing–review and editing. **Ying Tao**: writing–original draft, writing–review and editing. **Jiubo Fan**: writing–original draft, writing–review and editing. **Yuyu Zhou**: formal analysis, visualization, data curation, writing–review and editing. **Danyang Zhao**: visualization, formal analysis, writing–review and editing. **Yanlong Liu**: data curation, writing–review and editing. **Xiaoli Han**: conceptualization, methodology, funding acquisition, writing–review and editing. **Suriyakala Perumal Chandran**: conceptualization, methodology, writing–review and editing. **Fan Wang**: conceptualization, methodology, funding acquisition, writing–review and editing.

## Ethics Statement

The authors affirm that all procedures contributing to this study complied with the ethical standards of the relevant national and institutional committees on human experimentation, as well as with the Helsinki Declaration of 1975, revised in 2008. Ethical approval was granted by the Institutional Review Board of Inner Mongolian Medical University (approval number: YKD2014031). Written informed consent was obtained from all participants. No financial compensation was provided for participation. This study is not a clinical trial; therefore, a clinical trial registration number is not applicable.

## Conflicts of Interest

The authors declare no conflicts of interest.

## Peer Review

The peer review history for this article is available at https://publons.com/publon/10.1002/brb3.70784.

## Data Availability

The data that support the findings of this study are available from the corresponding authors. Requests to access these datasets should be directed to Fan Wang.

## References

[brb370784-bib-0001] Allard, C. , F. Bonnet , B. Xu , et al. 2019. “Activation of Hepatic Estrogen Receptor‐Alpha Increases Energy Expenditure by Stimulating the Production of Fibroblast Growth Factor 21 in Female Mice.” Molecular Metabolism 22: 62–70. 10.1016/j.molmet.2019.02.002. 30797705 PMC6437689

[brb370784-bib-0002] Alzheimer's Association . 2014. “2014 Alzheimer's Disease Facts and Figures.” Alzheimer's & Dementia 10, no. 2: e47–92. 10.1016/j.jalz.2014.02.001. 24818261

[brb370784-bib-0003] Ananya, F. N. , M. R. Ahammed , S. Lahori , et al. 2023. “Neuroprotective Role of Klotho on Dementia.” Cureus 15, no. 6: e40043. 10.7759/cureus.40043. 37425590 PMC10324629

[brb370784-bib-0004] Babcock, K. R. , J. S. Page , J. R. Fallon , and A. E. Webb . 2021. “Adult Hippocampal Neurogenesis in Aging and Alzheimer's Disease.” Stem Cell Reports 16, no. 4: 681–693. 10.1016/j.stemcr.2021.01.019. 33636114 PMC8072031

[brb370784-bib-0005] Banks, W. A. , S. A. Farr , T. S. Salameh , et al. 2018. “Triglycerides Cross the Blood‐Brain Barrier and Induce central Leptin and Insulin Receptor Resistance.” International Journal of Obesity 42, no. 3: 391–397. 10.1038/ijo.2017.231. 28990588 PMC5880581

[brb370784-bib-0006] Benoit‐Pilven, C. , J. V. Asteljoki , J. T. Leinonen , J. Karjalainen , M. J. Daly , and T. Tukiainen . 2025. “Early Establishment and Life Course Stability of Sex Biases in the human Brain Transcriptome.” Cell Genomics 5, no. 7: 100890. 10.1016/j.xgen.2025.100890. 40425010 PMC12278626

[brb370784-bib-0007] Bortz, J. , K. C. Klatt , and T. C. Wallace . 2022. “Perspective: Estrogen and the Risk of Cognitive Decline: A Missing Choline(rgic) Link?” Advances in Nutrition 13, no. 2: 376–387. 10.1093/advances/nmab145. 34849527 PMC8970832

[brb370784-bib-0008] Branigan, K. S. , and B. T. Dotta . 2024. “Cognitive Decline: Current Intervention Strategies and Integrative Therapeutic Approaches for Alzheimer's Disease.” Brain Science 14, no. 4: 298. 10.3390/brainsci14040298. PMC11048559 38671950

[brb370784-bib-0009] Cassader, M. , R. Gambino , G. Ruiu , S. Marena , P. Bodoni , and G. Pagano . 1996. “Postprandial Triglyceride‐Rich Lipoprotein Changes in Elderly and Young Subjects.” Aging: Clinical and Experimental Research 8, no. 6: 421–428. 10.1007/BF03339605. 9061130

[brb370784-bib-0010] Chen, L. , L. Fu , J. Sun , et al. 2023. “Structural Basis for FGF Hormone Signalling.” Nature 618, no. 7966: 862–870. 10.1038/s41586-023-06155-9. 37286607 PMC10284700

[brb370784-bib-0011] Ding, X. , J. Boney‐Montoya , B. M. Owen , et al. 2012. “BetaKlotho Is Required for Fibroblast Growth Factor 21 Effects on Growth and Metabolism.” Cell Metabolism 16, no. 3: 387–393. 10.1016/j.cmet.2012.08.002. 22958921 PMC3447537

[brb370784-bib-0012] Do, P. T. , D. M. Chuang , C. C. Wu , et al. 2024. “Mesenchymal Stem Cells Overexpressing FGF21 Preserve Blood‐Brain Barrier Integrity in Experimental Ischemic Stroke.” Translational Stroke Research 15, no. 6: 1165–1175. 10.1007/s12975-023-01196-8. 37783839

[brb370784-bib-0013] Dolegowska, K. , M. Marchelek‐Mysliwiec , M. Nowosiad‐Magda , M. Slawinski , and B. Dolegowska . 2019. “FGF19 Subfamily Members: FGF19 and FGF21.” Journal of Physiology and Biochemistry 75, no. 2: 229–240. 10.1007/s13105-019-00675-7. 30927227 PMC6611749

[brb370784-bib-0014] Drew, D. A. , R. Katz , S. Kritchevsky , et al. 2020. “Fibroblast Growth Factor 23 and Cognitive Impairment: The Health, Aging, and Body Composition Study.” PLoS ONE 15, no. 12: e0243872. 10.1371/journal.pone.0243872. 33306729 PMC7732072

[brb370784-bib-0015] Drew, D. A. , H. Tighiouart , T. M. Scott , et al. 2014. “FGF‐23 and Cognitive Performance in Hemodialysis Patients.” Hemodialysis International 18, no. 1: 78–86. 10.1111/hdi.12100. 24164913 PMC4443906

[brb370784-bib-0016] Falamarzi, K. , M. Malekpour , M. F. Tafti , N. Azarpira , M. Behboodi , and M. Zarei . 2022. “The Role of FGF21 and Its Analogs on Liver Associated Diseases.” Frontiers in Medicine 9: 967375. 10.3389/fmed.2022.967375. 36457562 PMC9705724

[brb370784-bib-0017] Farr, S. A. , K. A. Yamada , D. A. Butterfield , et al. 2008. “Obesity and Hypertriglyceridemia Produce Cognitive Impairment.” Endocrinology 149, no. 5: 2628–2636. 10.1210/en.2007-1722. 18276751 PMC2329289

[brb370784-bib-0018] Gomez‐Soria, I. , I. Iguacel , A. Aguilar‐Latorre , et al. 2023. “Cognitive Stimulation and Cognitive Results in Older Adults: A Systematic Review and Meta‐Analysis.” Archives of Gerontology and Geriatrics 104: 104807. 10.1016/j.archger.2022.104807. 36116285

[brb370784-bib-0019] He, Q. , Q. Li , J. Zhao , et al. 2016. “Relationship Between Plasma Lipids and Mild Cognitive Impairment in the Elderly Chinese: A Case‐Control Study.” Lipids in Health and Disease 15, no. 1: 146. 10.1186/s12944-016-0320-6. 27595570 PMC5011904

[brb370784-bib-0020] Hsuchou, H. , W. Pan , and A. J. Kastin . 2013. “Fibroblast Growth Factor 19 Entry Into Brain.” Fluids Barriers CNS 10, no. 1: 32. 10.1186/2045-8118-10-32. 24176017 PMC3818657

[brb370784-bib-0021] Jack, C. R. Jr. , R. C. Petersen , Y. C. Xu , et al. 1999. “Prediction of AD With MRI‐Based Hippocampal Volume in Mild Cognitive Impairment.” Neurology 52, no. 7: 1397–1403. 10.1212/wnl.52.7.1397. 10227624 PMC2730146

[brb370784-bib-0022] Kunert, S. K. , H. Hartmann , D. Haffner , and M. Leifheit‐Nestler . 2017. “Klotho and Fibroblast Growth Factor 23 in Cerebrospinal Fluid in Children.” Journal of Bone and Mineral Metabolism 35, no. 2: 215–226. 10.1007/s00774-016-0746-y. 27017221

[brb370784-bib-0023] Lane, C. A. , J. Hardy , and J. M. Schott . 2018. “Alzheimer's Disease.” European Journal of Neurology 25, no. 1: 59–70. 10.1111/ene.13439. 28872215

[brb370784-bib-0024] Lehallier, B. , D. Gate , N. Schaum , et al. 2019. “Undulating Changes in Human Plasma Proteome Profiles Across the Lifespan.” Nature Medicine 25, no. 12: 1843–1850. 10.1038/s41591-019-0673-2. PMC7062043 31806903

[brb370784-bib-0025] Li, H. , Z. Cao , J. Xu , et al. 2018. “Cerebrospinal Fluid FGF23 Levels Correlate With a Measure of Impulsivity.” Psychiatry Research 264: 394–397. 10.1016/j.psychres.2018.04.032. 29677623

[brb370784-bib-0026] Lin, W. , T. Zhang , Y. Zhou , J. Zheng , and Z. Lin . 2021. “Advances in Biological Functions and Clinical Studies of FGF21.” Diabetes, Metabolic Syndrome and Obesity 14: 3281–3290. 10.2147/DMSO.S317096. PMC8291585 34295169

[brb370784-bib-0027] Liu, P. , L. Chen , X. Bai , A. Karaplis , D. Miao , and N. Gu . 2011. “Impairment of Spatial Learning and Memory in Transgenic Mice Overexpressing Human Fibroblast Growth Factor‐23.” Brain Research 1412: 9–17. 10.1016/j.brainres.2011.07.028. 21824606

[brb370784-bib-0028] Liu, Y. , M. Wang , X. Tan , et al. 2017. “Negative Correlation Between Cerebrospinal Fluid FGF21 Levels and BDI Scores in Male Chinese Subjects.” Psychiatry Research 252: 111–113. 10.1016/j.psychres.2017.01.075. 28259034

[brb370784-bib-0029] Lu, J. , D. Li , F. Li , et al. 2011. “Montreal Cognitive Assessment in Detecting Cognitive Impairment in Chinese Elderly Individuals: A Population‐Based Study.” Journal of Geriatric Psychiatry and Neurology 24, no. 4: 184–190. 10.1177/0891988711422528. 22228824

[brb370784-bib-0030] Lu, J. , Z. Liu , Y. Zhao , X. Liu , W. He , and L. Zhang . 2023. “FGF19 improves Sevoflurane‐Induced Cognitive Dysfunction in Rats Through the PGC‐1alpha/BDNF/FNDC5 Pathway.” Tissue & Cell 81: 102012. 10.1016/j.tice.2022.102012. 36608639

[brb370784-bib-0031] Mahncke, H. W. , B. B. Connor , J. Appelman , et al. 2006. “Memory Enhancement in Healthy Older Adults Using a Brain Plasticity‐Based Training Program: A Randomized, Controlled Study.” PNAS 103, no. 33: 12523–12528. 10.1073/pnas.0605194103. 16888038 PMC1526649

[brb370784-bib-0032] Mao, H. Y. , H. C. Hsu , and S. D. Lee . 2020. “Gender Differences in Related Influential Factors of Regular Exercise Behavior Among People in Taiwan in 2007: A Cross‐Sectional Study.” PLoS ONE 15, no. 1: e0228191. 10.1371/journal.pone.0228191. 32004330 PMC6993962

[brb370784-bib-0033] Markan, K. R. 2018. “Defining “FGF21 Resistance” During Obesity: Controversy, Criteria and Unresolved Questions.” F1000Res 7: 289. 10.12688/f1000research.14117.1. 29983922 PMC6020717

[brb370784-bib-0034] McDicken, J. A. , E. Elliott , G. Blayney , et al. 2019. “Accuracy of the Short‐Form Montreal Cognitive Assessment: Systematic Review and Validation.” International Journal of Geriatric Psychiatry 34, no. 10: 1515–1525. 10.1002/gps.5162. 31243810

[brb370784-bib-0035] Minami, S. , S. Sakai , T. Yamamoto , et al. 2024. “FGF21 and Autophagy Coordinately Counteract Kidney Disease Progression During Aging and Obesity.” Autophagy 20, no. 3: 489–504. 10.1080/15548627.2023.2259282. 37722816 PMC10936614

[brb370784-bib-0036] Miyake, A. , and N. Itoh . 1996. “Rat Fibroblast Growth Factor Receptor‐4 mRNA in the Brain Is Preferentially Expressed in Cholinergic Neurons in the Medial Habenular Nucleus.” Neuroscience Letters 203, no. 2: 101–104. 10.1016/0304-3940(95)12272-9. 8834103

[brb370784-bib-0037] Nasreddine, Z. S. , N. A. Phillips , V. Bedirian , et al. 2005. “The Montreal Cognitive Assessment, MoCA: A Brief Screening Tool for Mild Cognitive Impairment.” Journal of the American Geriatrics Society 53, no. 4: 695–699. 10.1111/j.1532-5415.2005.53221.x. 15817019

[brb370784-bib-0038] O'Shea, A. , R. A. Cohen , E. C. Porges , N. R. Nissim , and A. J. Woods . 2016. “Cognitive Aging and the Hippocampus in Older Adults.” Frontiers in Aging Neuroscience 8: 298. 10.3389/fnagi.2016.00298. 28008314 PMC5143675

[brb370784-bib-0039] Rabin, L. A. , C. M. Smart , and R. E. Amariglio . 2017. “Subjective Cognitive Decline in Preclinical Alzheimer's Disease.” Annual Review of Clinical Psychology 13: 369–396. 10.1146/annurev-clinpsy-032816-045136. 28482688

[brb370784-bib-0040] Raz, N. , U. Lindenberger , K. M. Rodrigue , et al. 2005. “Regional Brain Changes in Aging Healthy Adults: General Trends, Individual Differences and Modifiers.” Cerebral Cortex 15, no. 11: 1676–1689. 10.1093/cercor/bhi044. 15703252

[brb370784-bib-0041] Sa‐Nguanmoo, P. , P. Tanajak , S. Kerdphoo , et al. 2016. “FGF21 improves Cognition by Restored Synaptic Plasticity, Dendritic Spine Density, Brain Mitochondrial Function and Cell Apoptosis in Obese‐Insulin Resistant Male Rats.” Hormones and Behavior 85: 86–95. 10.1016/j.yhbeh.2016.08.006. 27566237

[brb370784-bib-0042] Scheltens, P. , B. De Strooper , M. Kivipelto , et al. 2021. “Alzheimer's Disease.” Lancet 397, no. 10284: 1577–1590. 10.1016/S0140-6736(20)32205-4. 33667416 PMC8354300

[brb370784-bib-0043] Seidel, U. K. , J. Gronewold , M. Volsek , et al. 2014. “The Prevalence, Severity, and Association With HbA1c and Fibrinogen of Cognitive Impairment in Chronic Kidney Disease.” Kidney International 85, no. 3: 693–702. 10.1038/ki.2013.366. 24088956

[brb370784-bib-0044] Semba, R. D. , A. R. Moghekar , J. Hu , et al. 2014. “Klotho in the Cerebrospinal Fluid of Adults With and Without Alzheimer's Disease.” Neuroscience Letters 558: 37–40. 10.1016/j.neulet.2013.10.058. 24211693 PMC4037850

[brb370784-bib-0045] Sun, Y. , Y. Wang , S. T. Chen , et al. 2020. “Modulation of the Astrocyte‐Neuron Lactate Shuttle System Contributes to Neuroprotective Action of Fibroblast Growth Factor 21.” Theranostics 10, no. 18: 8430–8445. 10.7150/thno.44370. 32724479 PMC7381735

[brb370784-bib-0046] Taliyan, R. , S. K. Chandran , and V. Kakoty . 2019. “Therapeutic Approaches to Alzheimer's Type of Dementia: A Focus on FGF21 Mediated Neuroprotection.” Current Pharmaceutical Design 25, no. 23: 2555–2568. 10.2174/1381612825666190716101411. 31333086

[brb370784-bib-0047] Tan, B. K. , M. Hallschmid , R. Adya , W. Kern , H. Lehnert , and H. S. Randeva . 2011. “Fibroblast Growth Factor 21 (FGF21) in Human Cerebrospinal Fluid: Relationship With Plasma FGF21 and Body Adiposity.” Diabetes 60, no. 11: 2758–2762. 10.2337/db11-0672. 21926274 PMC3198100

[brb370784-bib-0048] Tang, M. , S. Cheng , L. Wang , et al. 2023. “Decreased FGF19 and FGF21: Possible Underlying Common Pathogenic Mechanism of Metabolic and Cognitive Dysregulation in Depression.” Frontiers in Neuroscience 17: 1165443. 10.3389/fnins.2023.1165443. 37266540 PMC10229787

[brb370784-bib-0049] Taniguchi, H. , Y. Hashimoto , N. Dowaki , and S. Nirengi . 2024. “Association of Brown Adipose Tissue Activity With Circulating Sex Hormones and Fibroblast Growth Factor 21 in the Follicular and Luteal Phases in Young Women.” Journal of Physiological Anthropology 43, no. 1: 23. 10.1186/s40101-024-00371-6. 39354624 PMC11446134

[brb370784-bib-0050] Tippen, S. P. , M. L. Noonan , P. Ni , et al. 2021. “Age and Sex Effects on FGF23‐Mediated Response to Mild Phosphate Challenge.” Bone 146: 115885. 10.1016/j.bone.2021.115885. 33618073 PMC8009839

[brb370784-bib-0051] Ursem, S. R. , C. Diepenbroek , V. Bacic , et al. 2021. “Localization of Fibroblast Growth Factor 23 Protein in the Rat Hypothalamus.” European Journal of Neuroscience 54, no. 4: 5261–5271. 10.1111/ejn.15375. 34184338 PMC8456796

[brb370784-bib-0052] Ursic‐Bedoya, J. , C. Chavey , G. Desandre , et al. 2022. “Fibroblast Growth Factor 19 Stimulates Water Intake.” Molecular Metabolism 60: 101483. 10.1016/j.molmet.2022.101483. 35367668 PMC9019402

[brb370784-bib-0053] Villarroya, J. , J. M. Gallego‐Escuredo , A. Delgado‐Angles , et al. 2018. “Aging Is Associated With Increased FGF21 Levels but Unaltered FGF21 Responsiveness in Adipose Tissue.” Aging Cell 17, no. 5: e12822. 10.1111/acel.12822. 30043445 PMC6156525

[brb370784-bib-0054] Wang, Q. , J. Yuan , Z. Yu , et al. 2018. “FGF21 Attenuates High‐Fat Diet‐Induced Cognitive Impairment via Metabolic Regulation and Anti‐Inflammation of Obese Mice.” Molecular Neurobiology 55, no. 6: 4702–4717. 10.1007/s12035-017-0663-7. 28712011 PMC5971086

[brb370784-bib-0055] Xia, X. , Q. Jiang , J. McDermott , and J. J. Han . 2018. “Aging and Alzheimer's Disease: Comparison and Associations From Molecular to System Level.” Aging Cell 17, no. 5: e12802. 10.1111/acel.12802. 29963744 PMC6156542

[brb370784-bib-0056] Yan, J. , Y. Nie , J. Cao , et al. 2021. “The Roles and Pharmacological Effects of FGF21 in Preventing Aging‐Associated Metabolic Diseases.” Frontiers in Cardiovascular Medicine 8: 655575. 10.3389/fcvm.2021.655575. 33869312 PMC8044345

[brb370784-bib-0057] Ye, L. , X. Wang , C. Cai , et al. 2019. “FGF21 Promotes Functional Recovery After Hypoxic‐Ischemic Brain Injury in Neonatal Rats by Activating the PI3K/Akt Signaling Pathway via FGFR1/Beta‐klotho.” Experimental Neurology 317: 34–50. 10.1016/j.expneurol.2019.02.013. 30802446

[brb370784-bib-0058] Zhai, W. , T. Zhang , Y. Jin , S. Huang , M. Xu , and J. Pan . 2023. “The Fibroblast Growth Factor System in Cognitive Disorders and Dementia.” Frontiers in Neuroscience 17: 1136266. 10.3389/fnins.2023.1136266. 37214403 PMC10196031

[brb370784-bib-0059] Zhang, X. , H. Zheng , Z. Ni , et al. 2024. “Fibroblast Growth Factor 21 Alleviates Diabetes‐Induced Cognitive Decline.” Cerebral Cortex 34, no. 2: bhad502. 10.1093/cercor/bhad502. 38220573 PMC10839844

[brb370784-bib-0060] Zhao, L. , H. Jiang , J. Xie , et al. 2022. “Effects of Fibroblast Growth Factor 21 on Lactate Uptake and Usage in Mice With Diabetes‐Associated Cognitive Decline.” Molecular Neurobiology 59, no. 9: 5656–5672. 10.1007/s12035-022-02926-z. 35761156

[brb370784-bib-0061] Zheng, P. , F. Wang , H. Li , et al. 2024. “Changes in Metabolic Hormones and Trace Elements in CSF in Active Smokers Indicate Oxidative Damage to Brain Cells.” Endocrine Connections 13, no. 6: e240016. 10.1530/EC-24-0016. 38688314 PMC11227062

[brb370784-bib-0062] Zhou, B. , K. E. Claflin , K. H. Flippo , et al. 2022. “Central FGF21 Production Regulates Memory but Not Peripheral Metabolism.” Cell Reports 40, no. 8: 111239. 10.1016/j.celrep.2022.111239. 36001982 PMC9472585

